# Student evaluation of teaching and assessment methods in pharmacology

**DOI:** 10.4103/0253-7613.64502

**Published:** 2010-04

**Authors:** Dinesh K Badyal, Suman Bala, Prashant Kathuria

**Affiliations:** Department of Pharmacology, Christian Medical College and Hospital, Ludhiana – 141 008, Punjab, India

**Keywords:** Evaluation, pharmacology, teaching

## Abstract

**Background:**

The students are in the best position to comment on the effectiveness of any teaching system and they may be regarded as the best judges to assess the teaching and evaluation methods.

**Objective:**

This study was designed to obtain student feedback on teaching and assessment methods in the subject of pharmacology and use it for improvement.

**Materials and Methods:**

Based on student feedback from batch 2006, innovative strategies were implemented. To know the effect of these strategies feedback was obtained from subsequent batch 2007 using a written validated questionnaire covering various aspects of teaching and assessment methods.

**Results:**

Students were satisfied with all teaching methods except lecture, seminars and pharmacy exercises. Majority of the students showed preference for tutorials, short answer questions and revision classes. All students felt that there should be more time for clinical pharmacology and bedside teaching. The performance score of the students (batch 2007) indicated improvement in their scores (12%) when earlier feedback suggestions were implemented. The pass percentage of the subsequent batch in university examinations improved from 90 to 100%.

**Conclusion:**

The implementation of suggestions obtained from students resulted in improvement in their performance. Hence, it is very essential to synchronize teaching and evaluation methods with special requirements of medical students.

## Introduction

An enhanced education of medical students in clinical pharmacology and therapeutics is important to ensure an effective and safe drug therapy. However, the pharmacology curriculum has not shifted much toward clinical pharmacology in India in undergraduate teaching. Attempts have been made all over India to make the teaching of pharmacology more interesting and relevant.[1] The course assessment instruments like feedback may help to know about the pros and cons of teaching and assessment methods. Currently, student's feedback represents the primary means used by most programs to assess their methodology.[2,3] Though a lot of verbal and non-verbal feedback is conveyed to the faculty in pharmacology, most of it is not published. This inspired us to undertake this study to evaluate the teaching and assessment in pharmacology for undergraduate medical students.

## Materials and Methods

**Figure 1:**
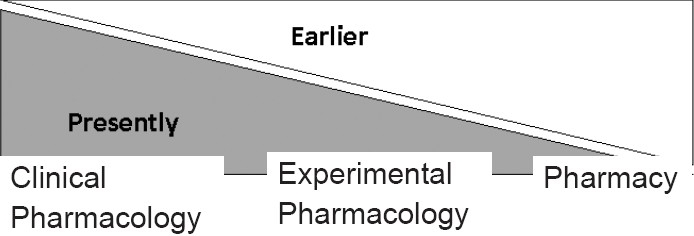
Shift in pharmacology teaching focus

The study was conducted at the Department of Pharmacology of Christian Medical College and Hospital, Ludhiana, among the students of second professional. Based upon an earlier feedback, we had reversed the trend of pharmacology teaching as shown in Figure 1. We tried to emphasize more on clinical pharmacology than the simple textbook knowledge and pharmacy exercises. The strategy implemented in a subsequent batch (batch 2007; n=50) is given in Table 1. The curriculum and the student characteristics were similar. A structured validated questionnaire was developed that consisted of 30 questions with three to five options. Students were also allowed to offer their own suggestions/remarks. Faculty of the department individually analyzed the frequency of different statements and then agreed on a common, consensus.

**Table 1:** The strategies implemented for learning and assessment

*Strategy*	*Teaching opportunities/areas*	*Assessment opportunities/areas*
Student seminars: The students were required to present seminars on a particular topic given to them. A faculty moderator was assigned and a time of 10 days was given for preparation.	Interactions with faculty for a longer time	Interaction with moderator
	Communication skills	Presentation
	Presentation skills	Participation in other seminars
	Confidence level	
	Handling queries	
The number of students was decreased in each tutorial group from 12 to 6-8. It was made sure that for consecutive four tutorial classes same moderator stayed with one batch.	To maintain continuity of the thought process and interaction of students with that particular moderator.	Knowledge gained Leadership qualities Synthesis, analytical skills
	Development of group dynamics skills	
Unnecessary animal experiments were replaced by computer simulation models (CSM).	With the implementation of CSM, we were able to save 3 months, in which we introduced more of clinically relevant exercises.	Interpretation of data Self assessment on free software
	Easy understanding of mechanism of action	
	Self-learning at any time	
The dispensing pharmacy exercises deleted.	Greater emphasis on clinical pharmacy, instructions to patients	Problem based exercises (e.g. tuberculosis case history) introduced
Formative assessment	Better learning opportunities as student knew that marks are not being used for summative assessment Students developed an attitude to learn	Day to day evaluation rather than a few tests in-between the course. All the tests were not included in calculating internal assessment.

## Results and Discussion

**Figure 2:**
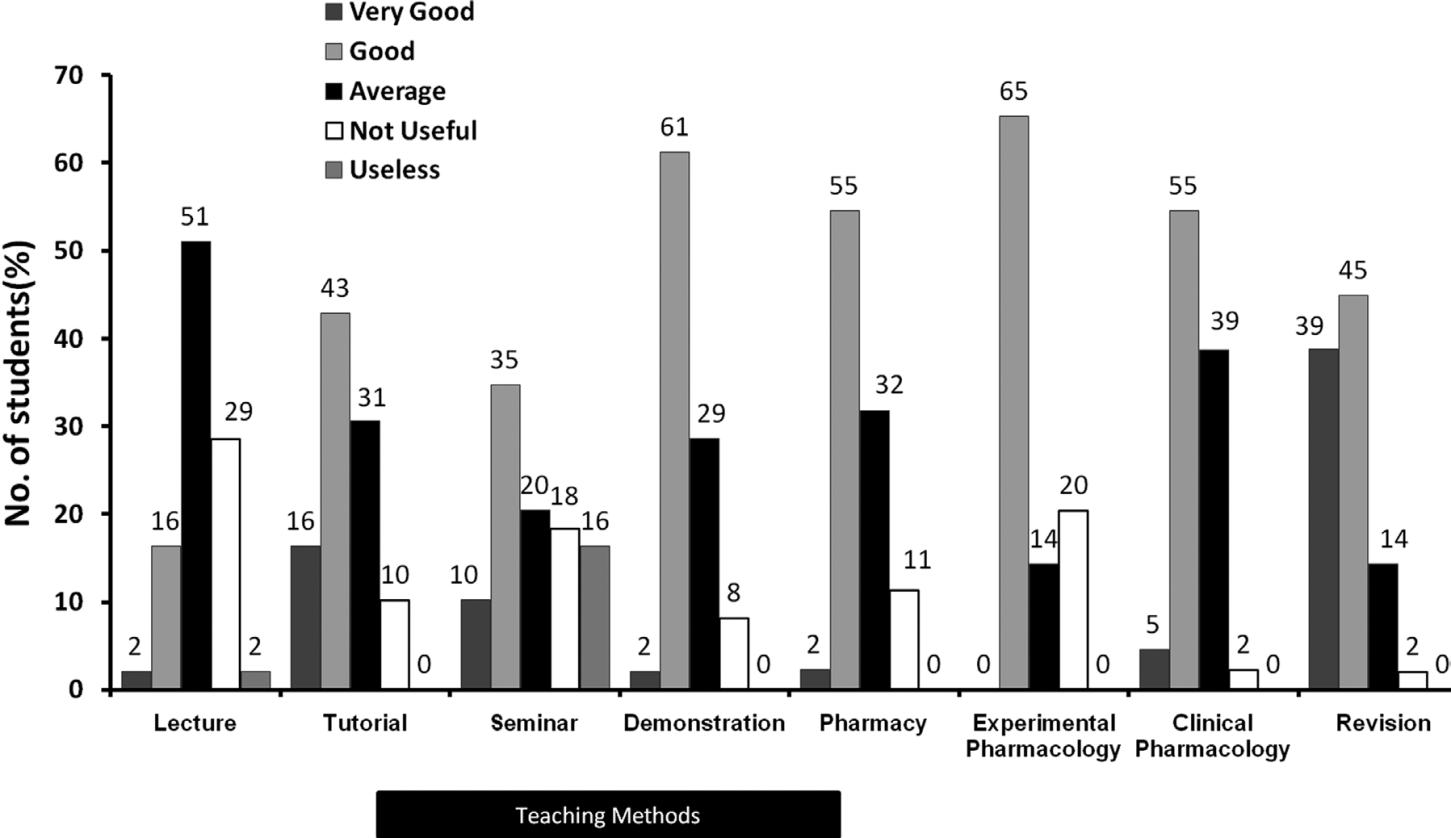
Feedback on teaching methods

A total number of 98% of the questionnaires were found to be sufficient for data analysis. Tutorials, demonstrations, experimental pharmacology, and revision classes were rated as good or very good by most of the students [Figure 2]. Students found revision classes held at the end of the course to be the best form of teaching (84%) followed by practical demonstrations and the practical work done by the students themselves (83%). Students found lectures to be an average or no utility. They emphasized that lectures should be more interactive. We noted that students are usually satisfied with interactive activities or teaching methods (e.g., tutorials, demonstrations, experimental pharmacology, and revision classes). Students expressed that animals should not be sacrificed for experiments and computer simulation models may be used instead. Students further added that pharmacy exercises should be decreased and should be restricted to clinical pharmacy only. They also opined for an increase in number of clinical pharmacology exercises and bedside teaching classes.

In assessment methodology, pre-university tests held at the end of course were found to be most useful (86%) in preparing for final university examination followed by class tests (84%), revision tests (81%), tutorials (63%), and seminars (35%). Students suggested that tutorials should follow a uniform pattern. Students did not favor seminars in the present study. This could be because they need to put in efforts to prepare for these. Few students (35%) felt that 18 months were enough to cover the pharmacology syllabus, but 60% felt that it can be shortened to 12-15 months. A majority of the students (45%) suggested that only few class tests should be taken into account for calculating internal assessment (IA) marks. Most of the students said that their IA marks were as per their performance throughout the course. The university examinations scores of the batch 2007 in which we implemented innovative methods showed significant improvement as compared to earlier batch (2006). The pass percentage of batch 2007 was 100% as compared to 2006 (90%). The average marks in pharmacology increased significantly by 12% in 2007.

In conclusion, it is important to know what our students need and whether they feel comfortable with the ever-expanding course with limited duration of time. Frequent feedbacks may help teachers plan the curriculum and improve upon the teaching and assessment methods. The limitation of the study was the bias among students or faculty participating in the study.
